# Carbon-free conferencing in the age of COVID-19

**DOI:** 10.1177/0030727020960492

**Published:** 2020-09-21

**Authors:** Linda JL Veldhuizen, Maja Slingerland, Lauren Barredo, Ken E Giller

**Affiliations:** 1 Plant Production Systems Group, 8225 Wageningen University, Wageningen, the Netherlands; 2 Sustainable Development Solutions Network (SDSN), New York, USA

**Keywords:** E-conference, online survey, Sustainable Development Goals, outreach, knowledge sharing, networking

## Abstract

The COVID-19 pandemic has been a crash course for many in working from home using various online tools, many of which can be used to organize e-conferences. An e-conference is a fully online event with multiple sessions and virtual discussion in one platform. In this paper, we aim to provide insights in and present key steps to organize a successful e-conference, increase our understanding of the impact of e-conferences, and identify key strengths, weaknesses, and success factors. Based on a participant survey and our own experience, we found that e-conferences are relatively easy to organize with readily-available and free tools, that they are more accessible and thus inclusive than physical meetings, and that they are virtually carbon-free which can contribute to large emission savings. Three important success factors are attracting a good set of speakers, building an interested audience, and reaching your objectives and desired impact. A successful e-conference can enable joint learning among speakers and participants, and allows novel ways of disseminating scientific knowledge while also enabling networking for the many participants who might not be able to attend an in-person event.

## Introduction

In response to the COVID-19 pandemic, working from home has become the new normal. Tools for online networking keep us connected with colleagues, while tools for hosting online meetings enable us to continue collaborating. While many large meetings and conferences were initially cancelled or postponed in the wake of the pandemic, organizers rapidly adopted the tools we use every day to accommodate large meetings online (Bhargava et al., 2020).

In 2018, long before the COVID-19 outbreak, the Sustainable Development Solutions Network’s Sustainable Agriculture and Food Systems group^1^ organized our first e-conference. An e-conference is in essence a fully online event with a platform with different spaces where multiple sessions and an online discussion can take place. In part it resembles a webinar, which also consists of an online session with one or more speakers. Two important differences, however, are that an e-conference consists of multiple online sessions, and that it, at every point possible, actively fosters and encourages interaction among participants and speakers via an online discussion platform where they can connect, discuss and share resources just as they would in a physical event. An e-conference is not merely a live broadcast of a physical event. Rather, it replicates the critical interactions that occur in physical events, both between speakers and participants as well as between participants, both during live sessions and between events in the series through an online conference platform. In our e-conferences all participants could directly engage in group discussions and/or choose to meet with other participants and speakers for bilateral discussions/virtual encounters mimicking corridor discussions in physical conferences. Its online format makes it more accessible than a physical event for both participants and speakers. Because speakers and audience members do not have to travel to the same physical location, there is more opportunity to attract a good selection of speakers within a short period of time as well as a diverse audience. As a result, e-conferences lend themselves to responding to urgent issues, and can be set up fast in response to emerging threats, such as fall armyworm in our case. In addition, its accessibility ensures that practitioners can quickly gain access to the latest scientific insights and apply them immediately in the field. Our e-conference was less theoretical than physical conferences (or MOOCS) and allowed for direct peer to peer exchange of experience in the discussion platform facilitating application of the knowledge provided and feedback on its results. A major reason for our decision to invest in e-conferences was to reach a much broader international audience, particularly people based in less-developed countries than is normally possible. This aligns with the ethos of the Sustainable Development Goals, ‘to leave no-one behind’.

An e-conference can take many forms depending on its objectives. If the objective is for scientists to exchange work and ideas, then the e-conference can take the form of a scientific conference with a scientific committee reviewing abstract submissions for poster and oral presentations (Hiltner, 2016). Such e-conferences have already been organized by several institutions (e.g. FAO, ICIMOD, USAID) since the late 1990s and their key outputs include conference proceedings of scientific contributions (e.g. Chandrasekharan, 1997; Keyser and Keyser, 2017; McGarry and Niino, 2011; Owen et al., 2011; Rana, 2003). If the objective of the e-conference is to engage and connect experts and practitioners around a specific topic, then the e-conference can take the form of a series of online keynotes with invited experts. For such events, outputs and impacts will need to be assessed differently. This type of e-conference is central in this paper.

When we organized our first e-conference, there were few examples of e-conferences, and our team learned ‘on the hoof’! We did not know what the best software environment would be, how long it would take to prepare, what potential pitfalls we needed to be mindful of, or what the potential impact might be. In this paper we therefore address three objectives: (1) to provide insights as to the key steps needed to organize a successful e-conference based on our experience (i.e. some do’s and don’ts); (2) to increase our understanding of the potential impacts of e-conferences for participants; and (3) to identify key weaknesses, strengths and success factors of e-conferences.

To meet these objectives, we evaluate our experiences gained from organizing and hosting four e-conferences, and we share results from a survey completed by participants in our first three e-conferences. We demonstrate that e-conferences are relatively easy and affordable to organize with readily-available tools, that they are more accessible and thus inclusive than physical meetings, and that they are virtually carbon-free, which can contribute to large emission savings.

## Materials and methods

From October 2018 to February 2020, we organized four e-conferences (Table 1) on various topics related to the work of the Sustainable Development Solutions Network (SDSN)’s Sustainable Agriculture and Food Systems group (SDSN, 2020a, 2020b, 2020c, 2020d). These e-conferences attracted up to 975 registrants and many of the session videos have since garnered several hundred views, reaching many more people than those attending the events live.

**Table 1.** Descriptive statistics for our four e-conferences (views as of 23 July 2020).

**E-conference**	**Number of registrations**	**Number of sessions**	**Number of views per video**
Fall armyworm in Africa, 22–26 October 2018	517	5	317–511
Nutrition-sensitive agriculture, 3–5 June 2019	975	3	499–1041
Fall armyworm in Asia, 10–12 July 2019	337	3	255–794
Resource recovery from sanitation, 19–21 February 2020	372	3	47–113*

* This e-conference could not be viewed live on YouTube like the other three e-conferences due to a change in software. Live session views are thus not included in this count as with the other three e-conferences.

We surveyed participants from the first three e-conferences to better understand their preferences and to determine the impact of these events. This survey ran from 21 November 2019 to 6 January 2020. It was sent out to 1,602 people, of whom 278 started and 254 finished the survey (a 16% response rate). We explored whether the population of respondents deviated from the overall population that participated in the webinar due to respondent self-selection.

The survey consisted of three parts. The first part concerned questions on the e-conference itself (after gauging which event(s) respondents attended), e.g. the preferred number of days for a virtual event, opinions on the session duration and number of speakers; opinions on Q&A during the live sessions; and opinions on the online conference platform. The second set of questions was intended to understand what kind of impact the e-conference had, how participants perceived it and how it compares to other forms of outreach by scientists. The third set of questions concerned basic demographics of the respondent such as country of residence, age and gender. A full copy of the survey can be found in the supplementary materials.

## Results

### Participation and participant characteristics in our four e-conferences and in the survey

Table 2 shows that the number of registrations per e-conference varied with the largest number of registrations for an e-conference with a globally-relevant topic, and the fewest number of registrations for an e-conference with a distinct regional perspective. The number of sessions, session duration and number of speakers per session varied among the e-conferences. In the first e-conference, most sessions had two to three speakers. Only the last session was comprised of short pitches on innovations and therefore had a larger number of speakers. Variation in the number of speakers was partly to test what worked best for moderators, speakers and the audience, but also due to practical considerations (e.g. the number of potential speakers who agreed to participate). There are programmatic advantages to both approaches; with one or two speakers, presentations and Q&A sessions can be longer. Conversely, involving more speakers reduces the time available for presentations, but allows for the inclusion of more topics or perspectives in one session.

**Table 2.** Descriptive statistics for our four e-conferences to date.

**E-conference**	**Number of sessions**	**Planned session duration**	**Number of speakers per session**
Fall armyworm in Africa, 22–26 October 2018	5	1½ hour	2–9
Nutrition-sensitive agriculture, 3–5 June 2019	3	1½ hour	1
Fall armyworm in Asia, 10–12 July 2019	3	1½ hour	2–3
Resource recovery from sanitation, 19–21 February 2020	3	2 hours	2–4

An online conference platform was set up for each of the four online conferences. Participants in the e-conference on ‘Fall armyworm in Asia’ were added to the already existing platform originally set up for participants in the e-conference on ‘Fall armyworm in Africa’. That platform was relocated a few months after the initial e-conference, but remained active. Participants were informed about the additional features of the platform when they received their confirmation of participation of the first life session, upon each new invitation for life sessions, during the life session when speakers pointed at material, at continued Q&A and at specific discussion points to find after the sessions. Participants could choose to receive alerts of new posts.

Table 3 shows that most posts were created during the e-conference on ‘Nutrition-sensitive agriculture’, followed by the e-conferences on ‘Fall armyworm in Asia’, ‘Fall armyworm in Africa’ and ‘Resource recovery from sanitation’. The number of views per post follows a similar pattern and aligns with the number of members on each platform. The proportionally larger number of views per post for the e-conference on fall armyworm in Asia is partly explained because the two e-conferences on fall armyworm shared the same platform. The relatively small number of posts for the e-conference on resource recovery from sanitation reflects the smaller number of active participants in the live sessions.

**Table 3.** Descriptive statistics on the online conference platforms set up for each e-conference.

	**Date platform went live**	**Number of members**	**Number of posts**	**Average number of views per post**
Fall armyworm in Africa*	5 April 2019	∼500	65	104
Nutrition-sensitive agriculture	27 May 2019	973	93	201
Fall armyworm in Asia*	8 July 2019	∼300	81	180
Resource recovery from sanitation	11 February 2020	370	16	140

* The two e-conferences on fall armyworm use the same online conference platform. The original online conference platform on fall armyworm in Africa (and all messages posted there) was lost after moving to a new location, which means that the actual number of messages that had been exchanged is larger than reported here.

Our software counted number of people logged in during life sessions, number of people registered for discussion platform, number of posts, number of views per post, but did not track individuals hence the same individual may contribute to more posts or views. In all e-conferences, the most frequent type of post was people introducing themselves to the community. The second most popular type of post on all platforms was to share resources, such as articles and reports. Other posts concerned questions, event announcements, polls, and general or other posts.

**Figure 1.**
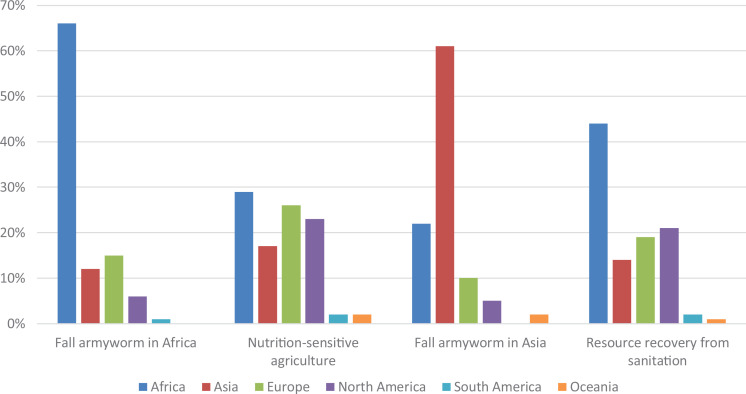
Participants’ continent of residence per e-conference.

The majority of participants in the regional e-conferences that specifically focused on Africa and Asia were from these regions (Figure 1). Participants in the e-conference on fall armyworm in Africa were from 60 countries, with most participants from Nigeria (62), Kenya (49) and Ghana (34). The e-conference on fall armyworm in Asia attracted participants from 56 countries, with substantial south-south learning and many participants from India (83), Vietnam (20) and the United States (16). The e-conference on nutrition-sensitive agriculture had a global perspective and attracted participants from 103 countries on all six continents. The countries with most participants were the United States (179), United Kingdom (52) and India (51). The e-conference on resource recovery from sanitation again had a global perspective and attracted participants from 79 countries. The three countries with the most participants were the United States (60), Nigeria (27) and Kenya (22).

Overall, substantial numbers of participants from the same countries, i.e. Nigeria, Kenya, India, and the United States attended the sessions. This in part aligns with 15% of survey respondents who indicated that they attended multiple e-conferences. In addition, these countries also represent where speakers were based, or where their work was focused.

**Figure 2.**
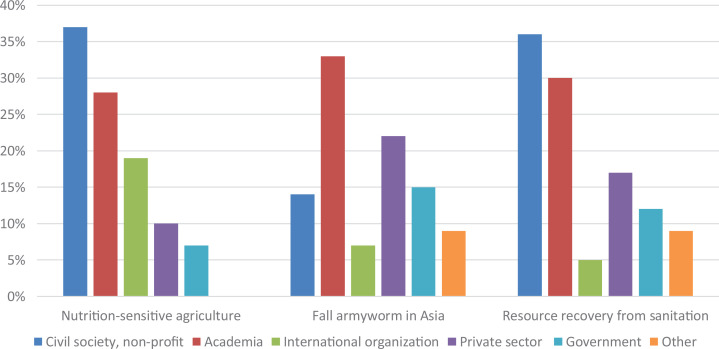
Participants’ sector of work per e-conference.

In all e-conferences, about a third of participants were from academia (Figure 2), as were the organizers and many of the speakers. This is perhaps unsurprising, as the main organizing institution, the SDSN, is a network of over 1,000 universities in over 100 countries and aggressively promoted the events to members. In the e-conferences on nutrition-sensitive agriculture and on resource recovery from sanitation, the largest share of participants were from civil society. In the e-conference on fall armyworm in Asia there were relatively fewer participants from civil society, while there were more participants from the private sector (Figure 2).

The survey sent to participants in the first three e-conferences had a response rate of 16%. The largest number of respondents had participated in the e-conference on ‘Nutrition-sensitive agriculture’ (102, or equal to 10% of e-conference participants), followed by the e-conference on ‘Fall armyworm in Africa’ (99, or 19% of participants). The largest share of e-conference participants responding to the survey attended the e-conference on ‘Fall armyworm in Asia’ (77, or 23% of participants). In total, the largest share of respondents was from Africa (43% responded to the survey, while they represented on average 39% of attendees across the e-conferences), followed by Asia (26% vs. 30%), Europe (15% vs. 17%), North America (10% vs. 11%), South America (2% vs. 1%) and Oceania (2% vs. 2%).

When participants registered for an e-conference, we did not ask for their age or gender, which unfortunately prevents us from comparing this to age and gender distributions in the survey to check for representativeness. Still, we can observe from the survey that the largest shares of respondents were aged 35–44 (34%), followed by 25–34 (31%), and 45–54 (18%). A larger share of respondents was male (57%), but we cannot ascertain whether this reflects the gender distribution in the e-conferences.

### Setting up an e-conference

Table 4 shows that an ideal timeline to set up and e-conference is 4–6 months, mainly depending on how long it takes to develop a programme and confirm speakers. Previous experience with the software means that less time is needed to select software and get familiar with it. We recommend starting event promotion approximately 4 weeks prior to the event. Nevertheless, in our experience the largest number of registrations will occur in the week prior to start of the e-conference.

**Table 4.** Timeframe for setting up an e-conference and steps involved based on the SDSN experience.

**Timing**	**Activity**
4–6 months in advance	Determine e-conference topic and objectives, set date and time, develop tentative programme and draft a list of potential speakers
4 months in advance	Contact potential speakers, choose software for the e-conference and develop a registration portal
3 months in advance	Develop a promotional kit and draft a list of contacts for targeted invitations
2 months in advance	Launch registration, send out invitations to contacts, set up the online conference platform and develop speaker instructions
4–6 weeks in advance	Finalize programme, send calendar invites to speakers and promote the e-conference (email, social media, etc.)
1–2 weeks in advance	Send e-conference information to participants (e.g. connection details, instructions on using the platform, etc.), launch online conference platform and organize a test session with speakers
Right before live session	Invite speakers to the live session, make sure everything works as it should and talk them through the programme
During the live session	Welcome participants, explain netiquette (e.g. mute microphones and cameras, procedure for asking questions) and programme (e.g. clarifying questions after the presentations, general discussion at the end), and introduce speakers
After live session	Ask presenters for permission to share PowerPoint presentations and continue the discussion on the online conference platform
After e-conference	Collate and share all e-conference materials with speakers and participants, as well as post publicly online

#### Programming

The first step in organizing an e-conference is to determine the topic. In determining your topic, it is important to have a clear audience in mind and to think of their (knowledge) needs. Clearly demarcate this topic to ensure that participant expectations are met. A timely topic that has not been addressed for your target audience will help to attract highly interested participants.

You also need to consider the duration of your e-conference. The majority of participants in our e-conferences indicated that they preferred an e-conference of 3 days or less (79%) with sessions of 60 minutes or less (62%) and two or three speakers (70%). A longer e-conference is possible, but you may want to consider spreading it out over more than 1 week to prevent fatigue of your audience, moderators and organizers. Despite what respondents indicated they prefer, we recommend either a maximum of two speakers for a 60-minute session or a longer session for more than two speakers. Interventions longer than 15 to 20 minutes are not recommended, as it is hard to keep a captive audience online; interaction and engagement are key to preventing drop-off. With two speakers in 60 minutes you will be able to give them sufficient time and attention, while also allowing at minimum one-third of the time for questions and answers (Q&A). A majority in our survey indicated they found the Q&A useful (85%) and would have liked more time for it (53%) with more interaction (63%).

When choosing the dates and times for your e-conference, some obvious considerations apply. Ensure that the conference does not coincide with important holidays for your target audience, and that the time of day is convenient for as many time zones as possible. If your audience is global, you could alternate with different times, although there will always be some who cannot join in real time. For this reason, it is important that video recordings become available as soon as possible after a session ends.

With your topic, objectives and audience in mind you can start developing a draft programme. This programme will help you identify potential speakers with a clear task, i.e. how would you like them to contribute? You will want to have a mix between more established names and younger contributors. An established name can give a great introduction to set the scene, while younger contributors can share recent and detailed insights from the field. Also, every effort should be made to ensure you have a mix of nationalities and good gender balance. When approaching speakers, it usually helps when you have a personal connection, even if an indirect one. If not, they are still likely to be interested, as most people enjoy talking about their work and engaging with an interested audience. Since there is no travel involved, you will not be asking for much of their time.

A critical factor to the success of the e-conference is how well it is promoted. There are many ways to promote your e-conference and it is wise to use all possible options, e.g. your contact list, newsletters, social media, discussion fora, etc. We found that the most effective outreach was via e-mail (48% of survey respondents indicated they heard about the e-conference in this way), followed by newsletters (13%), social media (with a slightly better reach for LinkedIn (9%) than for Facebook (7%) and Twitter (7%)), word-of-mouth (8%), and other (8%). We found that colleagues, speakers (plus their host institutions) and participants can be persuaded to help you promote the e-conference when you develop a promotional kit with suggested messages for the different channels.

#### Assuring that technology works

One important consideration is what software you will use for participant registration, the live sessions and the online conference platform. You may also use additional software to send out e-mails to your network, develop promotion materials, for the chat during the live sessions, etc. When choosing software, you should consider cost, features and functionality (e.g. is there a maximum number of participants, can anyone from any country easily access and use the software) and whether it generally works in a way that matches your needs.

Once you have determined which software you will use for your online conference platform you can start creating the first posts for the discussion forum. The very first post should explain how to use the different functionalities of the online platform. Other posts can ask people to introduce themselves, start discussions, or share resources and events. To get discussions started, it may be helpful to suggest specific threads, around key topics, geographic regions, etc. Once you have invited your participants to the platform, make sure to regularly visit it to moderate the discussion and create new posts, and encourage speakers to do the same.

Organizing an e-conference comes with its own set of challenges, mostly related to connectivity and software issues. Therefore, we highly recommend organizing a test session with all your speakers and moderators. Testing immediately before the event does not leave you time to troubleshoot any issues. Make sure you test camera and video quality, microphone (and muting), screen sharing, and any other functionalities you may want to use, such as playing video or polling. This test session not only serves to ensure that everything works but also to ensure that your speakers feel at ease with the software, to discuss the session agenda and address any questions they may have.

To make sure once more that everything works as it should, we recommend asking speakers to connect 30 minutes ahead of your scheduled start time. During the live session we recommend having two moderators; one to introduce the speakers, keep time, and moderate the discussion, and the other to monitor and moderate the live chat and incoming questions. You may also want to have a third person on stand-by to resolve any technical issues that may occur. After the session ends, share the video recording and slides (if speakers give permission) with participants. Once the e-conference has ended, collate all materials and share with participants and the broader public via social media, newsletters, and other outlets for those who missed the live event.

### Impact

A substantial share of survey participants (77%) indicated that they would not have been able to attend if the e-conference had been a physical rather than an online event. The reasons for this were the cost of travel (91%), the time it takes to travel (33%), visa challenges (24%), difficulty in getting supervisors to grant approval to attend (20%), climate concerns (15%), and health and family reasons (5%). Not having the funds to travel to conferences was the most important reason highlighted by respondents from all continents, although it was more prominent for respondents from Africa (94%) and Asia (95%) than from Europe (73%) and North America (69%). The second most important reason given by respondents from Africa was difficulties in obtaining a visa (27%). For respondents from Asia, not having the time (26%) or approval to attend (26%) were the most important reasons following cost. For respondents from Europe and North America, other important reasons were not having the time to travel (55% and 56%) and not wanting to travel due to climate concerns related to their personal carbon footprint (41% and 38%).

On a scale from 1 to 10 (with 1 indicating not interesting at all and 10 indicating very interesting), the e-conference on nutrition-sensitive agriculture received the highest average rating from participants (8), closely followed by the e-conferences on fall armyworm in Africa (7.8) and in Asia (7.7). The survey also included a number of statements to gauge elements of wider impact. For example, 95% of respondents indicated that they learned something new from the e-conference. Just under half of the respondents (47%) indicated that they met someone new, which could sometimes be seen on the e-conference platform when people exchanged contact details. Close to two-thirds of respondents (61%) indicated that they were able to apply the things they learned from the e-conference in practice. This illustrates that we achieved the objective of connecting experts and practitioners in order to put knowledge into practice. The impact of the event is even greater if we consider that 60% of respondents said they forwarded e-conference materials to others, indicating that more people used the knowledge that was shared in their daily practice.

E-conferences are a relatively new form of outreach that scientists can use to ensure that their knowledge and expertise reaches practitioners. Other tools for this are webinars, massive open online courses (MOOCs), conferences and seminars, articles and reports, workshops, and (social) media outreach. When asked to rank these forms of outreach from most preferred to least preferred, Table 5 shows that the more traditional forms of outreach (i.e. conferences and seminars, and articles and reports) are preferred over e-conferences. These forms of outreach are well-established (i.e. people know where to find and access them, and referring to these is widely accepted) and important for scientists’ careers. The respondents preferred e-conferences over webinars and MOOCs, two other forms of online outreach. One distinctive feature of e-conferences is the online conference platform where participants and speakers can interact with each other, which could explain why respondents ranked e-conferences over webinars. Our e-conference platform was used by approximately two-thirds of our respondents (64%). They mainly used it to view presenters’ slides (82%), read posts of others (69%), access video recordings (64%), connect with others (45%), get answers to their questions (37%), and to share materials (33%). Video and slides from the life sessions and the background material supporting it were made available immediately after the life session, whereas additional material was gradually provided upon demand, then gradually populated with on-demand materials. Many MOOCs have discussion platforms as well, but most emphasize an instruction (or top-down from teacher to student) structure of communication. Moreover, MOOCs require a more substantial time investment (several hours per week for several weeks) than webinars, e-conferences and workshops which may make them less attractive.

**Table 5.** Mean ranks for different forms of outreach by scientists.^a^

**Form of outreach**	**Average rank (on a scale of 1 to 7 with 1 being most desirable)**
Conferences and seminars	3.4
Articles and reports	3.6
E-conferences	3.7
Workshops	3.8
Webinars	3.9
MOOCs	4.7
Media and social media	5.0

^a^ Lower rank indicates a more desirable form of outreach.

## Discussion

We found that an e-conference is a valuable tool in a scientist’s toolbox for outreach. Compared with physical events it is more inclusive because there are virtually no barriers to join: no costs, a relatively small time investment, no visa required, and no emissions from travel. This is in line with advantages of other types of virtual conferences (Bhargava et al., 2020; Gichora et al., 2010) and webinar series (Fadlelmola et al., 2010). One barrier to inclusion that Ho et al. (2017) identified is that a time zone difference of more than 6 hours can lead to less participation. Indeed, time zone differences can prevent live participation, but this problem can be overcome through sophisticated IT solutions and repeated sessions (Gichora et al., 2010), or by offering the possibility to watch videos, access materials and participate in discussion platforms at any self-chosen time. There are also no ‘social’ barriers as people could access through an email or Facebook account and upon registration they did not have to leave additional information such as their affiliation, occupation, age, address or level of education. The only remaining barriers are that people need to have access to the internet and that they need to have received announcements or invitations to join. Still, the number of people who can access the internet is many times higher than the amount of people who can attend a physical meeting or workshop, access a scientific article, or extract relevant knowledge from a report, and this is especially true as more and more software options offer mobile apps to join by smartphone rather than computer.

A key strength of e-conferences is that it takes less time to organize because no time has to be allowed for abstract submission and selection. In addition, venues do not have to be secured, nor catering or travel arrangements made, which saves organizers a lot of time and money. Teams can also be much smaller as the key roles to fulfil are platform (technology) initiation and support, content lead and communication/moderation. As a result, an e-conference can be set up quickly in response to an emerging threat, such as fall armyworm or COVID-19, or to discuss the latest scientific insights. Compared with other forms of online outreach, notably webinars and MOOCs, e-conferences offer good opportunities for networking, require a manageable time investment and still results in effective dissemination of results.

A major benefit of e-conferences is that they do not require any of the participants or speakers to travel (except perhaps locally to a location with good internet connectivity). In the highly hypothetical situation that our first three e-conferences had been organized in Addis Ababa, Washington D.C. and New Delhi (the locations most central to likely participants and speakers, and with good international flight connections), and that all participants had travelled, the events would have produced 305, 802 and 183 metric tons of CO_2_ emissions, respectively. These potential emission savings, close to 1,300 metric tons of CO_2_ in total, is equal to 3.2 billion miles not driven by passenger vehicles, although we are not accounting for the emissions from using computers and accessing the internet to attend.

One potential challenge in organizing an e-conference is upholding the academic standards of peer review to ensure quality and for scholars or participants to earn credit (through references, conference proceedings, certificates of participation, etc.). It is possible to arrange systems and processes for this in a virtual format; however, careful consideration and planning of these systems will be needed from the outset to ensure effective engagement of all the necessary contributors. This would necessarily add to the planning timeline, and so careful consideration should be given as to whether or not peer review adds value to the event. In our case, our objective was to rapidly disseminate information to practitioners in the field, in order to respond to an urgent issue. Instituting a peer review process was not critical to the success of this endeavour, and instead our approach was to select well-respected and internationally-known speakers with a proven track record of sound science. Should you wish to go the alternative route, to date a number of publishers of scientific journals and books (Elsevier, Wiley, Taylor and Francis) leverage commercial search engines (such as Altmetric and PlumXmetrics) to evaluate the outreach of research output in social media, news outlets, blogs, etc. Furthermore, universities are revising their evaluation procedures to include such metrics, which are complementary to traditional, citation-based metrics. This will increasingly allow attributing credits to researchers that speak in e-conferences aimed at diverse audiences, without the need to go through prior peer-reviewed abstract evaluation. In a similar fashion, there are a growing number of analytics on conference platforms that allow organizers to track how long someone was logged on, how many comments they made, whether the meeting window was at the forefront of their screen, and more, to increasingly facilitate the accurate granting of certificates of participation, although it can be time consuming to conduct the necessary analyses and issue certificates, especially as virtual events have the potential to accommodate far larger numbers of participants than in-person meetings.

Three important success factors of an e-conference are attracting a good set of speakers (Fadlelmola et al., 2010), attracting an interested audience, and reaching your objectives and desired impact. Good speakers are not only the most established names in the field who can set the scene and provide an overview of existing knowledge. Earlier-career professionals can often offer more detailed and recent insights from the field (Gichora et al., 2010). It is not only important to have speakers of different ages and gender, but also to have speakers from different regions. When you have a specific geographical focus, it is important to have speakers from the region and speakers with experience in that region. When you have a global focus, you can still aim for speakers from different regions.

Attracting the right audience is another crucial success factor. A great set of speakers without an audience will not have an impact. To succeed, you need to know your audience and their knowledge needs, and you need to be able to reach them (Fadlelmola et al., 2010). We found that a key advantage of the online format is that it enables a global audience to participate. The main challenge, however, is to attract hard-to-reach groups, and in the case of our events, specifically farmers. Farmers in regions like Africa and Asia often lack access to the internet and are thus unable to join. Therefore, we conducted targeted outreach and marketing to people who work with farmers, such as extension workers, entrepreneurs, and key NGOs. We did notice, however, that our existing network of scientific contacts and peer institutions ensured a substantial audience from science. Reaching out to partners and networks outside our immediate domain allowed us to achieve our objective of attracting many practitioners.

The third success factor of an e-conference lies in its impact. It is important to define desired outcomes and impacts early in the planning process, and identify how you can track success, whether through qualitative or quantitative means. We identified four key questions to define success for our e-conference: were participants satisfied, did they learn something new and useful, did they make new connections, and was there interaction? We decided to do a post-event survey to determine whether or not the achieved these goals, and our survey found that respondents were generally satisfied with the three e-conferences. Perhaps even more importantly, almost all respondents indicated that they learned something new, that they were able to apply what they learned in practice and that they were able to expand their network. In addition, the videos continue to be viewed (sometimes by more people than originally subscribed to an e-conference) and the online discussion platforms are still in use today for networking and resource sharing.

This paper is based on lessons learned from organizing four e-conferences. Our experience alone would not have sufficed, which is why we sought the feedback of 1,600 participants to support and supplement our perspective as organizers. We were satisfied with the response rate and the survey’s representativeness across e-conferences and geographic regions. As with any survey, we expect those who enjoyed the e-conferences were more prone to take the survey. Still, we doubt this would have had a large influence on results, since most questions concerned preferences rather than appreciation. Much of the feedback from respondents confirmed our experiences. The only point of disagreement lies in the optimal session duration and the number of speakers. Respondents showed a preference for a shorter session with relatively many speakers and time for Q&A. We agree with the importance of including multiple speakers and allowing ample time for questions and discussion, which is why we would opt for a longer session duration.

The rise of COVID-19 has resulted in a sharp increase in the number of online meetings and events, and so almost every professional today has had a crash course in the use of online tools. At the same time, many of us have become overwhelmed by the large number of online meetings and events. Fatigue occurs when people have too many such meetings in a row without a break. Fatigue also occurs when meetings are poorly managed (Gichora et al., 2010), e.g. with no breaks during the meeting, little interaction between the audience and speakers, and static formats (e.g. long PowerPoint presentation) without much variation (e.g. an interview followed by a short film and then a panel discussion with input from the audience). Our e-conferences provided ample time for interaction in different ways such as Q&A, chats and polls during the sessions, and continuous discussions afterwards, which were all highly appreciated. The different modes of knowledge sharing (audio, video, graphs, discussions, debate, scientific papers, examples of life experiences) used in our e-conferences intended to increase inclusion and prevent drop-out as it aimed to resonate with participants different learning styles and different backgrounds and occupations. Everyone’s recent experiences with working online have started to show the need for such more engaging formats with sufficient time for breaks and offline work as well.

## Conclusion

Despite all the challenges that COVID-19 has brought, our hope is that the resulting rise in online meetings has opened the eyes of the scientific community to the possibilities and the advantages of e-conferences as outlined in this paper. The type of e-conferences discussed in this paper offer scientists a unique possibility to reach a large number of self-selected and hence highly interested participants from different backgrounds. Many of them would not have been reached otherwise, and based on our findings there is a justified expectation that many of them will use the knowledge presented in their work going forward, ensuring these events have a concrete impact. Additional advantages of e-conferences are that they are virtually carbon-free because they require no travel, and can be an affordable alternative to an in-person meeting. The e-conferences that were central to this paper were relatively easy to set up and can thus be organized quickly in response to an emerging topic for which there is a clear need to discuss the latest scientific insights and exchange experiences.

## Supplemental material

Supplemental Material, Appendix_1_-_Survey - Carbon-free conferencing in the age of COVID-19 Click here for additional data file. Supplemental Material, Appendix_1_-_Survey for Carbon-free conferencing in the age of COVID-19 by Linda JL Veldhuizen, Maja Slingerland, Lauren Barredo and Ken E Giller in Outlook on Agriculture

## Note

1.
https://www.unsdsn.org/sustainable-agriculture-and-food-systems.
