# Supplementary material for: Carbon-free conferencing in the age of COVID-19
Source: Outlook Agric. 2020 Sep 21;49(4):321–9. doi: 10.1177/0030727020960492 (PMC7684527; doi:10.1177/0030727020960492)
Supplement: Supplemental Material, Appendix_1_-_Survey - Carbon-free conferencing in the age of COVID-19 [file Appendix_1_-_Survey.pdf]

Thank you for agreeing to take part in this survey.

We invited you to this survey because you registered for one or more of our SDSN e-conferences. We would like to learn from your experiences to improve our future e-conferences.

This survey will take about 10 minutes and your responses will be treated confidentially.

Which e-conference(s) have you registered for?

- Responding to Fall Armyworm in Africa, 22-26 October 2018
- The Latest Evidence on Nutrition-Sensitive Agriculture, 3-5 June 2019
- Responding to Fall Armyworm in Asia, 10-12 July 2019
- None of the above [automatic redirect to the end of the survey]

How did you hear about our e-conferences?
You can select multiple options [randomized order]

- E-mail invitation
- Newsletter
- Twitter
- LinkedIn
- Facebook
- Word-of-mouth
- Other, please specify:

Our e-conferences differed in the number of days with a daily live session (3-5 days), the duration of these live sessions (60-120 minutes) and the number of speakers per live session (1-5 speakers).

Please indicate what would your ideal e-conference would look like [drop-down menu]:

- Number of days:
  - 1 day
  - 2 days
  - 3 days
  - 4 days
  - 5 days
  - more than 5 days
- Session duration per day:
  - 30 minutes
  - 60 minutes
  - 90 minutes
  - 120 minutes
  - more than 120 minutes
- Number of speakers per day:

- 1 speaker
- 2 speakers
- 3 speakers
- 4 speakers
- more than 4 speakers

Each live session ended with a discussion with the presenters based on your questions (Q&A).

Which of these statements about this Q&A applies to you?

	Yes	No	Not applicable / I’m not sure
I found the Q&A useful			
I would have like more time for the Q&A			
It was easy to ask my question			
I got an answer to my question			
I would have liked to more actively join the discussion			

Did you use our online conference platform Mobilize?
You can select multiple options

- No, I did not use the online conference platform
- Yes, I shared materials such as reports, upcoming webinars etc.
- Yes, I read posts of others
- Yes, I viewed the slides of the presenters
- Yes, I accessed the video recordings
- Yes, I connected with others
- Yes, I got answers to my questions
- Other, please specify:

If you have any suggestions to improve our online conference platform, please enter them here:

How would you rate the e-conference(s) you attended?
1 indicates not interesting at all, 10 indicates very interesting

	1	2	3	4	5	6	7	8	9	10
Responding to Fall Armyworm in Africa										
The Latest Evidence on Nutrition-Sensitive Agriculture										
Responding to Fall Armyworm in Asia										

Which of these statements about the e-conference(s) applies to you?

	Yes	No	I’m not sure
I learned something new			
I met someone new			
I was able to apply the things I learned in practice			
I invited someone else to the e-conference			
I forwarded e-conference materials to others			

Is there a barrier for you to join an event like this e-conference ‘in the real world’ (i.e. not online)?

- No
- Yes, I don’t have time to attend in person
- Yes, I don’t have the funds to attend
- Yes, it’s difficult for me to get a visa
- Yes, I don’t want to travel because of climate concerns
- Yes, I don’t want to travel due to health or family reasons
- Yes, I may not get permission to attend
- Other, please specify:

Please rank the following forms of outreach by scientists from most to least informative [options are shown in random order, people can drag and drop them in order]

- Webinars
- Massive Online Open Courses (MOOCs)
- Conferences and seminars
- Articles and reports
- Workshops
- Media and social media
- E-conferences

Where do you live?

- Africa
- Asia
- North America
- South America
- Europe
- Oceania

What areas do you focus on in your work? [multiple options are allowed]

- Africa
- Asia
- North America
- South America
- Europe
- Oceania
- Global

- No specific area focus

Which of these job titles best describes your current job? [options shown in random order]

- Farmer
- Extension worker
- Input supplier
- Breeder
- Processor
- Retailer
- Policy maker
- Researcher
- Teacher
- Student
- Nutrition officer
- Project manager
- Consultant
- None of the above, namely:

How old are you:

- 18-24
- 25-34
- 35-44
- 45-54
- 55-64
- 65+
- Prefer not to say

What is your gender?

- Male
- Female
- Prefer not to say
- Prefer to self-describe:

Please enter your comments, suggestions for improvements or suggestions for e-conferences topics here:
